# The effects of water lubrication of tracheal tubes on post-intubation airway complications: study protocol for a randomized controlled trial

**DOI:** 10.1186/s13063-016-1699-0

**Published:** 2016-11-25

**Authors:** Eugene Kim, Seong Mi Yang, So Jeong Yoon, Jae-Hyon Bahk, Jeong-Hwa Seo

**Affiliations:** 1 Department of Anesthesiology and Pain Medicine, Seoul National University Hospital, Seoul National University College of Medicine, 101 Daehak-ro, Jongno-gu, Seoul, 110-744 Republic of Korea; 2 Present Address: Department of Anesthesiology and Pain Medicine, Catholic University Hospital of Daegu, School of Medicine, Catholic University of Daegu, Daegu, Republic of Korea

**Keywords:** Airway management, Anesthesia, General, Intratracheal, Intubation, Lubrication, Water

## Abstract

**Background:**

Water is known to have lubricating properties, thus it is used for lubrication of tracheal tubes to reduce airway injuries caused by intubation. However, there is no definite evidence to substantiate the beneficial effects of lubricating tracheal tubes using water for attenuating airway injuries. Moreover, the lubrication pretreatment may cause contamination of the tube, leading to respiratory infections. Therefore, this trial aims to assess whether no pretreatment of tracheal tubes does not increase post-intubation airway complications as compared with water lubrication of tubes.

**Methods/design:**

This is a prospective, double-blind, single-center, parallel-arm, noninferiority, randomized controlled trial to be conducted in participants aged 20–80 years who are undergoing elective surgery under general anesthesia with orotracheal intubation. Participants are randomly assigned into one of two groups depending on whether intubation is performed using a tracheal tube lubricated with water (*n* = 150) or without any pretreatment (*n* = 150). The primary outcome is the incidence of sore throat at 0, 2, 4, and 24 h after surgery, which is analyzed with a noninferiority test. The secondary outcomes are the incidence and severity of postoperative hoarseness, oropharyngeal injuries, and respiratory infections.

**Discussion:**

Because we hypothesized that lubricating tracheal tubes using water has no advantage in reducing airway injuries associated with intubation, we will compare the incidence of sore throat, which is the most common complaint after intubation, in a noninferiority manner. This is the first randomized controlled trial to investigate the possibly beneficial or harmful effects of lubricating tracheal tubes using water before intubation. We expect that this trial will provide useful evidence to formulate a protocol for preparing tracheal tubes before intubation.

**Trial registration:**

This trial is registered at ClinicalTrials.gov on 1 July 2015 (NCT02492646)

**Electronic supplementary material:**

The online version of this article (doi:10.1186/s13063-016-1699-0) contains supplementary material, which is available to authorized users.

## Background

Water is commonly used for lubrication of various medical devices, including tracheal tubes, because it is known to have lubricating properties [1–6]. However, some other lubricants have shown no beneficial effects on reducing airway injuries associated with tracheal intubation such as sore throat or hoarseness [7–9]. Moreover, there has been no evidence as to whether the lubrication of tracheal tubes using water decreases airway injuries caused by intubation, so this conventional pretreatment seems to have been performed without any validation for its advantage.

Besides, tracheal tubes are known to be a major source of respiratory infections in patients undergoing mechanical ventilation [10–13]. Moreover, the wet condition of the tube may aggravate the proliferation of respiratory pathogens, thereby increasing the risk of infection [14]. Any external treatment applied to the tracheal tube before intubation can lead to contamination of the tube as opposed to keeping the tube inside the sterile packing until intubation. Thus, lubricating tracheal tubes using water may even be harmful, increasing the probability of respiratory infection.

Therefore, we conducted a prospective, randomized noninferiority trial to investigate whether no pretreatment of tracheal tubes does not increase post-intubation airway complications as compared with water lubrication of tubes in patients undergoing general anesthesia. We also examine postoperative respiratory infections to evaluate the adverse effects of water lubrication of tracheal tubes.

## Methods/design

### Study design

**Fig. 1:**
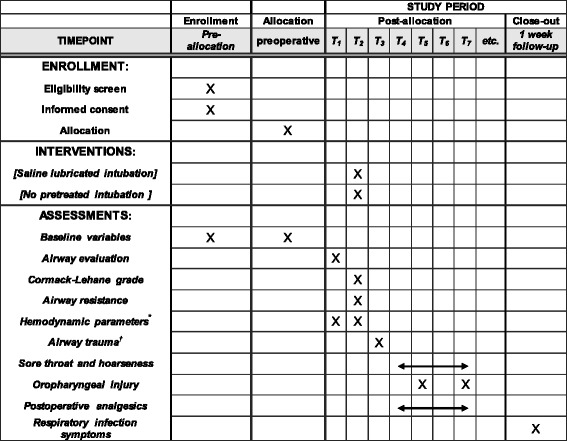
Standard Protocol Items: Recommendations for Interventional Trials (SPIRIT) flow diagram: schedule of enrollment, interventions, and assessments. *: mean blood pressure, heart rate, and pulse oximetry, †: examination of the presence of blood on the tube surface or in the oral cavity; T_1_: before intubation, T_2:_ intubation, T_3_: emergence period, T_4_: at post-anesthesia care unit, T_5_: postoperative 2 h, T_6_ : postoperative 4 h, T_7_: postoperative 24 h

This prospective, double-blind, single-center, parallel-arm, noninferiority, randomized controlled trial was approved by the Institutional Review Board of Seoul National University Hospital (version 2.0, reference number: 1506-125-684, validated on 28 July 2015) and registered at ClinicalTrials.gov (NCT02492646). This trial is being conducted in a tertiary university hospital in Seoul, South Korea. We describe the protocol of our study according to the Standard Protocol Items: Recommendations for Interventional Trials (SPIRIT) guidelines (Fig. 1 and Additional file 1). The final report of this protocol will follow both the general Consolidated Standards of Reporting Trials (CONSORT) Statement as well as its extension to noninferiority trials.

### Participants

Potential participants who meet the inclusion criteria are recruited at outpatient clinics or on the preoperative visit 1 day before surgery, and written informed consents are obtained from all of the participants (by SJY). We enroll participants with American Society of Anesthesiologists physical status 1–3 and aged 20–80 years, who are scheduled for elective surgery under general anesthesia with orotracheal intubation. The exclusion criteria are as follows:

- Symptoms of sore throat, hoarseness, and respiratory infections as assessed by a study investigator at baseline
- Gastroesophageal reflux diseases defined by history-taking from patients
- Congenital or acquired abnormalities of the upper airway
- Previous airway surgeries
- Previous history of aspiration
- Coagulation disorders
- History of difficult intubation or conditions with an expected difficult airway including Mallampati classification ≥3 or a thyromental distance <6.5 cm
- Use of airway instruments other than a direct laryngoscope such as a fiberoptic bronchoscope, video laryngoscope, or lighted stylet
- Anticipated nasotracheal intubation or insertion of a nasogastric tube

### Randomization and blinding

**Fig. 2:**
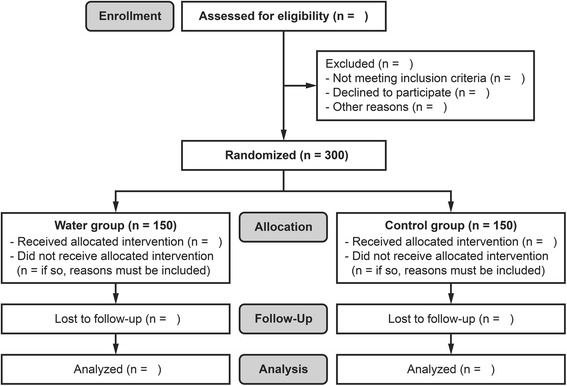
Consolidated Standards of Reporting Trials (CONSORT) flow chart

After the recruitment, participants are randomly assigned to one of the two groups in a 1:1 ratio depending on whether or not the tracheal tube is lubricated with water before intubation (Fig. 2). A random sequence with 4 or 6 sizes of random blocks (i.e., 4-4-4-6-4-4-6-6…) is generated with an online tool (http://www.randomization.com/) by an assistant not involved in the trial and kept within sealed opaque envelopes. When a patient is enrolled in the trial, an anesthesia nurse opens an envelope and prepares a tracheal tube with the allocated treatment.

All participants, outcome assessors except for the intubation practitioner (EK), and data analysts are blind to treatment allocation. The unique identification number, which is linked to the randomization schedule, will be managed by an assistant who does not participate in the study. If serious adverse events that threaten the safety of participants (such as death or irreversible injury to the respiratory system) occur, we will immediately stop the intervention, cancel the blinding, and contact the Institutional Review Board.

### Withdrawal, dropout, and discontinuation

Participants can refuse to participate in the study and follow-up evaluation after enrollment. Other indications for withdrawal are the following situations: when tracheal intubation is performed by methods other than direct laryngoscopy, such as using a fiberoptic bronchoscope, video laryngoscope, or lighted stylet, and when extubation is not achieved after surgery. If the planned statistical power is not obtained because of a high dropout rate, additional recruitment will be done according to a new randomization.

### Confidentiality

Unnecessary individual data including name, social security number, or chart number of participants will not be collected throughout the study period. Only the study code and phone number of the participants will be collected and managed separately. Collected data will be kept confidential until the limited investigators analyze the data. After completion of the study, the collected data will be stored encrypted for 3 years and then discarded.

### Intervention

According to random allocation, a disposable tracheal tube (Unomedical, Kedah, Malaysia), which is made of polyvinyl chloride and has a cuff with high-volume and low-pressure characteristics, is pretreated by an anesthesia nurse unaware of the study protocol. Until tracheal intubation, the tube is placed in a 1-L bottle of sterile saline for the experimental group or kept inside the sterile packing for the control group. Tubes with 7.0-mm and 7.5-mm internal diameters are used for women and men, respectively.

After overnight fasting, participants enter the operating room without any premedication. With standard monitoring including electrocardiography, noninvasive arterial blood pressure, and pulse oximetry general anesthesia is induced with intravenous administration of propofol 1.5–2.0 mg/kg and fentanyl 1 mcg/kg. Rocuronium 0.6–0.8 mg/kg is administered for the neuromuscular blockade and train-of-four counts are checked at the adductor pollicis muscle using acceleromyography (TOF-watch®, Organon Ltd., Dublin, Ireland). At a train-of-four count of 0, the tracheal tube, pretreated according to the randomization, is handed to an investigator (EK) who has a 10-year experience in tracheal intubation. The investigator performs tracheal intubation via direct laryngoscopy using either Macintosh 3 or 4 blades. If intubation fails, the tube tip is flexed to make a hockey-stick shape by inserting the stylet inside the tube, and then intubation is reattempted. When intubation fails even using the stylet, other devices such, as a video laryngoscope, fiberoptic bronchoscope, or lighted stylet, are applied. After successful intubation, the intracuff pressure is adjusted to less than 25 cm H_2_O using a cuff pressure monitor (VBM Medizintechnik GmbH, Sulz am Neckar, Germany) [15].

Anesthesia is maintained with 1.0–1.5 minimum alveolar concentration of desflurane to obtain a bispectral index (A-2000 XP, Aspect Medical Systems, Newton, MA, USA) value of less than 60, and total fresh gas flow is supplied at 2 L/min throughout the operation. All participants receive inhalation with nonhumidified anesthetic gas through a disposable semiclosed breathing circuit using an anesthetic machine (Avance, GE Datex-Ohmeda, Munich, Germany). Mechanical ventilation is performed with a tidal volume of 6–8 ml/kg and a positive end-expiratory pressure of 5–10 cm H_2_O. The respiratory rate and inspired oxygen fraction are adjusted to maintain an end-tidal carbon dioxide partial pressure of 30–40 mmHg and oxygen saturation by pulse oximetry of 95–100%, respectively.

At the end of surgery, pyridostigmine 0.3 mg/kg and glycopyrrolate 0.01 mg/kg are administered to antagonize the neuromuscular blockade. After gently suctioning the oral secretions from the oropharynx, extubation is carefully performed at a train-of-four ratio above 90% when the participants are able to achieve spontaneous breathing and obey verbal commands.

### Measurements

During direct laryngoscopy, the laryngeal view is classified using the Cormack-Lehane classification [16]: grade 1, no difficulty; grade 2, only posterior extremity of the glottis visible; grade 3, only the epiglottis visible; and grade 4, no recognizable structures visible without laryngeal manipulation. The intubation practitioner subjectively evaluates the resistance during advancement of the tube through the glottis using a four-point scale (none, mild, moderate, and severe). Hemodynamic parameters including noninvasive blood pressure, heart rate, and pulse oximetry are recorded immediately before and 1 min after intubation. Intubation time, defined as the duration between the insertion of the laryngoscopic blade into the mouth and the inflation of the endotracheal tube cuff, is measured. Extubation time is defined as the interval from cessation of anesthesia until extubation. After extubation, the presence of blood on the tube surface or in the oral cavity is examined.

An investigator (SMY) blinded to the group assignment evaluates patient-reported sore throat and hoarseness 0, 2, 4, and 24 h after surgery. The severity of sore throat is evaluated using a four-point scale as follows: none, no sore throat; mild, complained of sore throat only upon inquiry; moderate, complained of sore throat without inquiry; severe, change of voice or hoarseness associated with throat pain [17]. Hoarseness is defined as a subjective symptom that is different from the previous voice quality of the patient [18]. The investigator also examines oropharyngeal injuries via direct inspection using a penlight and tongue depressor with regard to the location (posterior pharyngeal wall, uvula, tonsillar fossa, pillar, or others) and type (hyperemia, edema, hematoma, or others) at 2 and 24 h after surgery. The amount of intraoperative and postoperative analgesic drugs is checked until 24 h after surgery.

At 7 days after surgery, the investigator also asks about the symptoms of respiratory infections, such as cough, sputum, rhinorrhea, sore throat, or fever, and whether the patients have been diagnosed with a common cold, tonsillitis, pneumonia, or any other respiratory infectious diseases and prescribed related medications postoperatively. If the patients have been discharged from hospital, they are contacted by telephone and asked about respiratory infections.

### Sample size

The primary outcome of this trial is the incidence of postoperative sore throat within 24 h after surgery. Assuming the incidence of 57% in a previous study [17], 135 patients are required in each group to obtain 80% statistical power, 5% risk of type-I error, and 15% noninferiority margin. The margin was determined based on our clinical judgment that the incidence of postoperative sore throat is relatively high (50–60%), so the difference within 15% in the incidence would be considered clinically noninferior. Considering 10% dropout, the estimated sample size is 300 in both groups. The sample size is calculated using a PASS software (version 11.0, NCSS, Kaysville, UT, USA).

### Statistical analysis

Both intention-to-treat and per-protocol analyses will be performed and missing data will be imputed with the last observation carried forward value. Continuous variables will be presented as mean and standard deviation or median and interquartile range according to the Kolmogorov-Smirnov test, and categorical variables as number of patients and proportion.

The incidence of postoperative sore throat (primary outcome) will be compared with a noninferiority analysis. The noninferiority of the nonpretreated tube over the water-lubricated tube will be accepted if the upper bound of a 95% confidence interval is below the predetermined noninferiority margin of 15%. For secondary outcomes, continuous variables will be compared with an independent *t* test or the Mann-Whitney *U* test and categorical variables with Pearson’s chi-squared test or Fisher’s exact test. All tests are two-sided and *P* < 0.05 is considered statistically significant. A statistician not involved in data collection will conduct all statistical analyses using SPSS software (version 21.0, SPSS Inc., IBM, Chicago, IL, USA).

## Discussion

Lubrication of tracheal tubes is performed to reduce airway damage caused by tracheal intubation although it requires an extra procedure for preparation. Nevertheless, in previous studies several lubricants, such as lidocaine [7, 8] or hydrocortisone [9], failed to show beneficial effects on attenuating airway injuries associated with intubation. Moreover, lubrication of tracheal tubes inevitably increases the risk of contamination as compared with no pretreatment. Therefore, we conduct this prospective, randomized, double-blind trial to obtain evidence regarding the beneficial or harmful effects of lubricating tracheal tubes using water.

Sore throat is the most common complaint after tracheal intubation with its incidence reported as being up to 90% [4, 15, 19]. We thought that if lubricating tracheal tubes using water was effective in reducing airway injuries caused by intubation, it may primarily affect the incidence of postoperative sore throat, so we consider this as the primary outcome of our trial. We evaluate sore throat at several time points because it is known to occur within 24 h after surgery with varying incidence and severity [17, 20]. Because we hypothesized that lubricating tracheal tubes using water would have no advantage for reducing postoperative sore throat, we will compare its incidence in a noninferiority manner. We also examine hoarseness and oropharyngeal injuries to obtain more evidence for airway injuries associated with intubation [21–23].

Lubricating tracheal tubes using water might be effective for attenuating airway injuries, but it can increase the chance of contamination and infection. Moreover, water may aggravate the proliferation of respiratory pathogens, worsening the infections [14]. Therefore, we evaluate the clinical symptoms of respiratory infections within 7 days after surgery.

To the best of our knowledge, this is the first randomized controlled trial to investigate the probable advantage and disadvantages of lubricating tracheal tubes using water, which has been conventionally performed for the pretreatment of tracheal tubes for intubation. Therefore, we expect that this trial may provide useful evidence to formulate a protocol for preparing tracheal tubes before intubation.

### Trial status

The recruitment commenced in August 2015 and aims to enroll 300 participants for the trial. It is anticipated that recruitment will end by January 2017.

## Additional file

Additional file 1: SPIRIT Checklist. recommended items to address in a clinical trial protocol and related documents. (PDF 128 kb)
